# What Is Disease? What Diagnosis?

**Published:** 1907-10-19

**Authors:** Sydney W. Macilwaine

**Affiliations:** (retired), Peterhurst, Cleveden, Somerset.


### SPECIAL ARTICLE.

### WHAT IS DISEASE? WHAT DIAGNOSIS?

By SYDNEY W. MACILWAINE, M.R.C.S., L.R.C.P. (retired), Peterhurst, Cleveden, Somerset.

### II. MEDICINE, SURGERY, AND SPECIALISM IN RELATION TO SCIENCE AND ART.

(*Concluded from page* 36.)

The ultimate object of all our work, as a profession, is a purely practical one; therefore there is a tendency to try to reach practical results without wasting time over theory, the speediest possible cure is generally sought. But the art of medicine is an applied science, and success in its exercise must ultimately depend on the perfection of the pure science on which it is founded. Science is organised knowledge of Nature, and of this great whole medical science is a branch; it is organised knowledge of human disease. There is no question of exact or inexact science here; the ebb and flow of human disease is, like the ebb and flow of the tides that wash our shores, part of the invariable, natural sequence of cause and effect. All inexactness in science is the result of ignorance or of the failure to record events accurately.

The relation of science and art in medicine is analogous to that ruling in any other applied science: one man guides a ship to the antipodes, another vaccinates against smallpox; both found their practice on pure science, on organised knowledge of Nature. In describing the pure science of medicine, the causes of disease, both intrinsic and extrinsic, were set out, and the ultimate goal of medical science was shown to be the complete correlation of cause and effect in the whole range of human disease, the definite linking of all symptoms with their cause or causes, for of course the same symptoms occur in many diseases.

The object and scope of the art that will be founded on this science are clear. Science teaches that every cause of disease is natural, and, therefore, discoverable; art learns from this that no cause of disease, once discovered, is altogether beyond control, and that therefore the time has come when the whole art of medicine must be based primarily on prevention. In the past, treatment was practically synonymous with cure and alleviation; as soon as it is realised that the correlation of cause and effect is always possible, our invariable rule must be to seek to deal with causes instead of being content to tinker at effects. The scientific investigation of every case involves a double problem, the tracing of effect to cause—the making of a diagnosis—and the elucidation of the conditions leading to each attack of disease. Here again it is clear that, once fully understood, these conditions may be always more or less modified.

The whole situation may be very simply stated by saying that if we know exactly why certain symptoms develop, our treatment of the individual attacked will be rational, and we shall be in the best possible position to forestall the symptoms altogether in other cases. The unification and organisation of our science would inevitably and immediately lead to the placing of the prevention of disease, the preservation of health, in its proper position of pre-eminence in our art.

The bearing of this proposition on the present condition of our work is evident; the practical importance of deciding what we mean by a disease and its diagnosis is clear. However lightly the "merely theoretical" question whether the "organic diseases" of morbid anatomy and the "diseases" of the eye, the ear, the skin, the heart, the nerves, the liver, or the mind are true or spurious may be dismissed, the fundamental importance of the question becomes immediately apparent in the light of practical application.

If diseases are to be labelled at the will of every specialist as synonymous with symptoms and to be regarded when convenient as belonging to organ, region, tissue, or function, diagnosis must be divided among a band of experts whose number is still growing, and treatment must of course follow the same plan, but if our science is to be unified, if diagnosis is to rest on the correlation of cause and effect, then treatment must be placed on the same broad basis. It must be added that if the meaning I have sought to attach to diagnosis is the true one, then not only is one half of our science chaotic because of the failure to recognise the intrinsic causes of disease, but it is also impossible to put the management of these diseases on a scientific basis until a knowledge of the patient's constitution (his intrinsic tendencies) is recognised as essential.

Again, I would insist that there is no room for argument in the ordinary sense of the word; these questions must be approached and answered without regard to personal or temporary considerations—there is a principle involved. Infantile paralysis, Menière's disease, Paget's disease, Hodgkin's disease, Hebra's prurigo, gout, diabetes, chorea, cancer, sarcoma, are only a few of the scores of symptom-groups of indeterminate causation that the student is taught to "diagnose," and what of treatment? I do not feel competent to say precisely what is empiricism, but we are all agreed that it ought not to be deliberately practised by the regular medical man.

Every practitioner will always relieve symptoms by any means in his power when he can do so safely; to relieve symptoms in typhoid fever or pneumonia is not empiricism, but the "purely expectant treatment" of infantile paralysis—whatever it may be—is an example of deliberate empiricism that has official sanction. False theory implies more or less futile practice; the student who is taught that he has made a diagnosis when he has detected infantile paralysis, or any other spurious disease, and that on this he must found his treatment, is taught empiricism of the avoidable variety. If every practitioner were taught that his scientific work is incomplete until he has traced effect to cause, that unless he can do this he is treating a patient for symptoms, not for a disease, his mind would be raised out of the beaten track of a dead routine; not only would be become an interested and useful worker in the field of the natural history of human disease, but his practice would become more intelligent and therefore safer and more effective.

The whole practice of medicine must be based on theory, as a house is based on foundations. A unified science would give a foundation for a broad, comprehensive art, and one mind might grasp both; on the other hand, if division and sub-division of the science of medicine is right, let this be done thoroughly on a definite plan, so that the whole profession shall understand and appreciate it.

My object in describing the science of medicine is to bring home to the mind of the medical man the fact that for all those who pursue this science—and this we all do throughout our professional life—there is one goal, the complete clinical correlation of cause and effect. From this the logical deduction is that the science of medicine is essentially indivisible, and that the same scientific obligation rests on everyone, call himself what he will, who takes charge of a case. This unity of the science implies breadth and comprehensiveness in the art that is founded on it; the complete correlation of cause and effect is the ideal of the scientific worker, the preservation of health is the goal of the same man when he seeks to apply his knowledge in practice. The question is, how nearly our present system approaches the ideal, and whether the two may be more nearly approximated.

Our work is supposed to be divided, roughly, into medicine, surgery, and specialism; the question that concerns us is whether this division is legitimate, and, if so, on what principle it is made. It has been here laid down as a fundamental principle that diagnosis—the tracing of effect to cause—is the primary duty of every regular practitioner, and that diagnosis must precede treatment. This brings us back to where we started, to the question, What is the meaning of diagnosis? I shall not repeat myself further than to say that if the specific word "disease" has two or more meanings, then there may be two, or twenty, different kinds of diagnosis of various value; and sub-division, specialism, in the pure science of medicine may be justified; but under no other circumstances can this be done. If, on the other hand, there is but one true meaning of the specific word "disease"; and if therefore every attack of every disease must be investigated in accordance with the same plain scientific method, then the whole of our art must be made candidly dependent on this unified science. The position is really so clear that I shall simply state my view of it, and leave those responsible to say for what reason they hold other views.

### The Art of Medicine.

The specific word "disease" has only one legitimate meaning, it represents cause and effect; diagnosis has only one true value, it implies the tracing of effect (symptoms) to cause; the obligation of diagnosis is the same for every regular practitioner. When the purely scientific part of our work is completed, when a diagnosis has been made, this knowledge is applied in treatment. It is clear that when a diagnosis has not been or cannot be made the obligation to recognise this candidly is the same for all. The line between diagnosis and treatment is always plain, and it is just as clear that the art of medicine is divisible as that the science is indivisible. This means that everyone who takes charge of a patient must be a diagnostician, and must share the common scientific obligation; but that once a diagnosis is made, treatment may possibly be handed over to an expert. Once it is realised that every true disease represents cause and effect and that, therefore, to speak of organic, surgical, special, or local "diseases" is a misuse of terms, it is easy to realise that surgery and all the minor specialisms are really sub-divisions of the art of medicine. We must return to the simpler ways of our forefathers and recognise again that the physician, the diagnostician, is the master of the situation; every attempt, conscious or unconscious, to escape from the scientific obligations of our profession is doomed to failure.

### Legitimate Specialism.

It is outside my present purpose to discuss the proper limits of legitimate specialism; it is clearly not only convenient, but necessary to specialise the operating surgeon in populous places, in the school, and in the great hospital. But this in no sense justifies the divorce of medicine from surgery, of art from science; when surgery and medicine were perversely separated as distinct callings, the septic infections raged unchecked in the surgical wards; the surgeon did not concern himself with diagnosis, and the physician passed by on the other side. But when Lord Lister correlated cause and effect in these diseases he not only proved himself one of the greatest of physicians, but he swept away the last relics of what another great surgeon calls an "unholy divorce." He showed that the modern surgeon is indeed an operating physician who interferes with special knowledge, and therefore with justified courage. In the matter of narrower specialism it seems to me clear on the one hand that definite sub-divisions of our art may be allotted to the dental and the obstetric surgeons with excellent results; but that, by contrast, the setting apart of a specialist for the "diagnosis and treatment" of the "diseases" of the skin is founded on palpable error and leads only to confusion and stagnation of knowledge.

It concerns every member of the profession, and not least the practitioner, to make up his mind whether the division and sub-division of our science among specialists is a chimera, or whether it holds the promise of the future. If this question were decided, the sub-division of our art in accordance with scientific requirements and special circumstances might be easily done, but the principle underlying the problem has, so far as I know, never been clearly and authoritatively laid down; it is to this omission that I wish to draw the attention of the profession.

